# Using community-based system dynamics modeling to understand the complex systems that influence health in cities: The SALURBAL study

**DOI:** 10.1016/j.healthplace.2019.102215

**Published:** 2019-11

**Authors:** Brent A. Langellier, Jill A. Kuhlberg, Ellis A. Ballard, S. Claire Slesinski, Ivana Stankov, Nelson Gouveia, Jose D. Meisel, Maria F. Kroker-Lobos, Olga L. Sarmiento, Waleska Teixeira Caiaffa, Ana V. Diez Roux, SALURBAL Group

**Affiliations:** a Department of Health Management and Policy, Dornsife School of Public Health, Drexel University, Philadelphia, PA, USA; b Department of Health Policy & Management, Gillings School for Global Public Health, University of North Carolina, Chapel Hill, NC, USA; c Social System Design Lab, Brown School, Washington University in St. Louis, St. Louis, MO, USA; d Urban Health Collaborative, Dornsife School of Public Health, Drexel University, Philadelphia, PA, USA; e Departamento de Medicina Preventiva, Faculdade de Medicina da Universidade de São Paulo, São Paulo, Brazil; f Facultad de Ingeniería, Universidad de Ibagué, Ibagué, Colombia; g INCAP Research Center for the Prevention of Chronic Diseases, Institute of Nutrition of Central America and Panama, Guatemala City, Guatemala; h Facultad de Medicina, Universidad de Los Andes, Bogotá, Colombia; i Observatório de Saúde Urbana de Belo Horizonte, Faculdade de Medicina, Universidade Federal de Minas Gerais, Belo Horizonte, Brazil

**Keywords:** Latin America, Diet, Transport, Community-based system dynamics, Group model building

## Abstract

We discuss the design, implementation, and results of a collaborative process designed to elucidate the complex systems that drive food behaviors, transport, and health in Latin American cities and to build capacity for systems thinking and community-based system dynamics (CBSD) methods among diverse research team members and stakeholders. During three CBSD workshops, 62 stakeholders from 10 Latin American countries identified 98 variables and a series of feedback loops that shape food behaviors, transportation and health, along with 52 policy levers. Our findings suggest that CBSD can engage local stakeholders, help them view problems through the lens of complex systems and use their insights to prioritize research efforts and identify novel solutions that consider mechanisms of complexity.

## Highlights

• CBSD methods can promote systems thinking and help identify policy approaches.
• Stakeholders identified 98 variables driving food behaviors and transport in cities.
• Balancing and reinforcing feedbacks drive change in food behavior and transport.
• Feedback loops can also explain policy responses and lack of policy responses.
• Stakeholders identified 52 policies that leverage variables and feedbacks.

## 1 Introduction

Over half of the world's population, more than 4.1 billion people, lives in cities (World Bank, 2015). In general, many health outcomes are better among urban populations than rural populations (Bai et al., 2012), although much heterogeneity exists both between and within cities. Many factors associated with urbanization can be good for health (e.g. greater employment opportunities, higher income levels, and access to more robust educational and health care systems), but others can be detrimental (e.g. sedentary lifestyles, consumption of processed foods, crowding, exposure to air pollution, and social inequalities) (Bai et al., 2012). Important questions remain regarding the dynamic processes that drive the health of urban residents (Diez Roux, 2015; Glouberman et al., 2006), as well as the policies and interventions that may be most effective in promoting the health of urban residents and the environmental sustainability of cities (Devi, 2015).

Latin America is among the world's most urbanized regions, with over 80% of people living in cities (World Bank, 2015). Many cities in Latin America have implemented innovative public health (e.g., sweetened beverage taxes, food labeling) and urban planning policies (e.g., bus rapid transit, cycling infrastructure) to improve health, sustainability, and quality of life (Colchero et al., 2016, 2017; Corvalán et al., 2013; Heinrichs and Bernet, 2014; Ramos et al., 2017). The unique policy environments within these cities can serve as a model for identifying approaches to improve the health and sustainability of cities across the globe, particularly those in low- and middle-income countries.

Two influential mechanisms through which city living impacts health are via factors related to food and transport systems. Despite living further from the places where food is produced, urban dwellers typically have access to a wide variety of healthy and unhealthy foods in restaurants, supermarkets, small corner stores, and other retailers (Fleischhacker et al., 2011; Richardson et al., 2012). Higher incomes coupled with greater access contribute to increased consumption of animal products, sugar, and processed foods among city dwellers in countries undergoing urbanization, which has fueled increases in obesity and chronic disease (Drewnowski and Popkin, 1997; Popkin, 1999). Other pathways through which urban environments likely affect diet include via city policies (e.g., food labeling, retailer restrictions), increased exposure to food marketing, and diet-related social norms, knowledge, and attitudes (Popkin, 1999, 2006). Pathways through which transport impacts the health of city residents include effects on active transport, sedentary behaviors and physical activity, commuting times, air pollution exposures and traffic related injuries (Frank and Engelke, 2001; Mueller et al., 2017). Urban planning decisions and urban transportation policies can impact health through their effects on automobile use, congestion, public transportation quality and availability, land use mix and pedestrian or cyclist– oriented development (Giles-Corti et al., 2016).

The multiple, interacting pathways through which cities impact health can be thought of as a complex adaptive system (Bai et al., 2012; Diez Roux, 2015). Among the hallmarks of a complex adaptive system are factors at multiple levels of influence (e.g., individual-, neighborhood-, and policy-level factors) and feedback loops. For example, consider the variables that impact city residents' choice of commute mode. The decision to commute via private vehicle or public transit may be influenced by city-level factors such as congestion, adequacy of the road network, and access, quality, and affordability of public transit (Cervero, 2002). The decision may also be influenced by interpersonal factors –active transportation methods like walking and cycling may be seen as more normative among individuals with friends and colleagues who commute via these modes (Bopp et al., 2013; Campbell and Bopp, 2013). Personal factors, including an individual's socio-demographic characteristics (e.g., income, car ownership) and commuting context (e.g., distance) may also influence their choice (De Witte et al., 2013).

Many of the factors that influence population health are likely to be interrelated and to influence each other via feedback loops, which make these systems adaptable. For example, consider a city with a transit network that includes roads, a bikeway, public transportation and transit oriented development. Under the initial conditions of the city, commuters will choose a transit mode based on context (e.g., relative prices), their commute characteristics (e.g., time by commute mode, distance, elevation change, neighborhood walkability and mixed land use), and preferences (e.g., attitudes towards bicycling). As the city's population grows, additional commuters may use the road network and urban sprawl may lengthen commuting distance. As a result, the city's road network will become more congested and driving times will increase. This change in driving times may cause some commuters who drive to re-evaluate their mode choice and switch to an alternative mode that is not as affected by congestion, like public transportation or cycling. As more and more people switch, the system will evolve a new state with a new distribution of drivers, cyclists, and public transportation users. This is an example of a balancing feedback loop, a type of stabilizing feedback structure that helps to regulate the effects of changes (i.e., population growth that adds new commuters) imposed on a system (Meadows, 2008). These complex dynamics of feedback and delays result in structures that may resist policy intervention or that may respond to policies in counterintuitive or counterproductive ways (Sterman, 2006). Identifying the feedback loops that impact people's behaviors can help us better understand why a complex system acts the way it does and can help build support for policy interventions to achieve healthier outcomes.

Few studies have explicitly employed systems approaches to inform our understanding of drivers of health in cities. However, there are a range of methods available to help describe and explore the structure and function of complex adaptive systems that include both formal simulation modeling and qualitative approaches. Formal models can help us understand how urban policies can be used to improve health, particularly when the pathways through which the policy achieves its effects are fairly well understood (Beran et al., 2019; Rutter et al., 2017). For example, promotion of walking and cycling for transport can reduce vehicle emissions and congestion in cities, as well as promote active lifestyles among city dwellers. However, these benefits must be weighed against the costs of increased exposure to air pollution among pedestrians and cyclists (Giles and Koehle, 2014; Giles-Corti et al., 2016). An alternative approach to reducing congestion and emissions might be to improve public transportation infrastructure and implement options like bus rapid transit in combination with transit oriented development together with transit oriented development (Cervero and Dai, 2014). Formal models can help to quantify the costs and benefits that might be expected under these alternative approaches and serve as a policy decision support tool.

Group model building is an approach to understanding how stakeholders perceive the variables, relationships, and feedback loops that comprise a complex adaptive system to co-create qualitative causal maps and system dynamics simulation models (Hovmand, 2014; Hovmand et al., 2015b). Community based system dynamics modeling (CBSD) prioritizes building stakeholders’ capabilities in the use of tools from the field of system dynamics (P Hovmand, 2014). Group model building and CBSD have been shown to improve understanding of a problem, promote systems thinking, and lead to consensus for action (Andersen et al., 1997; Hovmand, 2014; Richardson and Andersen, 1995; Vennix, 1996). The methods have been used across diverse disciplines (e.g., business, public health) in the public and private sector, and are well-suited to generate insights about the complex systems that drive health in cities (Brennan et al., 2015; Hovmand, 2014; Rouwette et al., 2002).

### 1.1 Participatory community-based system dynamics in Latin America

SALURBAL (Salud Urbana en América Latina) is a multi-country, interdisciplinary research team spanning 14 institutions and 8 countries across Latin America and the U.S. that is studying how urban environments and policies impact health, health equity, and environmental sustainability in Latin American cities (Diez Roux et al., 2018). One of the aims of SALURBAL is to use the tools of complex systems to gain insights into the complex and interrelated drivers of health in Latin American cities, and to identify effective policy levers. To address this aim, SALURBAL is using complex systems simulations (i.e., agent-based models) combined with a series of CBSD workshops with a diverse group of regional stakeholders. These workshops were broadly focused on understanding the key variables and feedback loops via which food behaviors and transport impact health and environmental sustainability in Latin American cities, and on identifying food and transport policies that could be used to improve health and the environment. We chose to concentrate on the food and transport systems based on the large role that diet and physical activity play in driving the burden of chronic disease in Latin American cities (Gakidou et al., 2017), and because they represent policy areas of particular interest to the region (LAC-Urban Health and SALURBAL, n.d., November LAC-Urban Health and SALURBAL, n.d.). In this study, we describe the objectives, design, and implementation of the study, present key evidence regarding achievement of each objective, and reflect on insights generated throughout the process.

## 2 Methods

### 2.1 Training and workshop design

The design process for the three CBSD workshops started with a 2.5-day training and planning session that brought together substantive experts from the research team with methodological experts on the use of CBSD. The session used a combination of lectures and hands-on experiential exercises to introduce key principles in system dynamics modeling, as well as the use of structured workshops and scripted activities (Hovmand et al., 2015b). As an example, a common script used in workshops is “Initiating and Elaborating a Causal Loop Diagram,” in which participants work together to draw causal maps that illustrate feedback loops that explain a dynamic behavior of interest. In the training session, participants elaborated two separate causal maps conveying their perspectives on traffic injuries and active transport. These activities were used to generate initial insights about these issues from a feedback perspective and motivate a discussion around how to adapt these scripts for use in workshops with stakeholders who had experience and deep knowledge of food and transportation systems in Latin American cities.

At the end of the training session, the team developed a list of explicit (i.e., communicated to participants prior to workshops) and implicit (i.e., values and principles that influenced the workshop approach) objectives to inform the design of the workshops (see Table 1), identified team members to serve key roles on the core modeling teams (CMTs), and laid out a framework for the design process. The CMTs consisted of a representative from the country hosting the workshop, at least one modeler, someone with knowledge of the participants, someone familiar with the objectives of the study, and at least one expert in CBSD methods. The CMTs for each workshop were responsible for convening over the two months preceding each workshop to select and adapt scripts, develop a facilitation manual for each workshop, work with team members from relevant countries to identify participants, and communicate on an ongoing basis with the larger SALURBAL study team and systems working group. In order to build capacity within our team (Objective E2), facilitation was led by members of the research team with substantive expertise in food and transport systems, and team members with methodological expertise in CBSD served as process coaches (please see Scriptapedia for descriptions of roles) (Hovmand et al., 2015a).

**Table 1:** Objectives of community-based system dynamics workshops.

Explicit objectives communicated to participants prior to the workshops
E1: Bring diverse stakeholders into an initiative to promote healthy, equitable, and sustainable cities in Latin America
E2: Gain experience in application of systems approaches in urban health problems and use of causal loop diagrams to identify and explore policy options
E3: Participants will provide input that will help identify and prioritize research questions and practice implications to be pursued by the SALURBAL project using systems modeling in the future

*Implicit objectives communicated to participants after the workshops*
I1: Put health and health equity on the agenda of policymakers who may not think their work influences health.
I2: Learn about and expand mental models (i.e., a cognitive representation of a real dynamic system) of stakeholders (academia, policymakers, banks, civil society) around transportation, food systems, and health
I3: Identify policy priorities for improving health through transportation and food system intervention and learn what is of value to stakeholders
I4: Identify common structures/drivers and variations across cities/contexts, and determine whether outputs of these workshops can inform the development of a simulation model
I5: Assess and test the waters for a potential simulation model/systems approach and dissemination beyond academia

### 2.2 Participant identification and recruitment

Between November 2017 and May 2018, the SALURBAL team conducted one CBSD workshop in each of three cities: Lima, Peru; São Paulo, Brazil; and Antigua Guatemala, Guatemala. In general, participants were purposively identified by SALURBAL team members from countries in the same region where each workshop was to be held (e.g., the Lima workshop included participants from Peru, Argentina, Chile, and Brazil). Participants were also targeted for recruitment based on their expertise in either food or transport systems. Within these content areas, three types of participants were targeted: elected and administrative policymakers, members of civil society (e.g., nonprofits), and academics. Additionally, two private sector stakeholders with relevant domain expertise were identified by Brazilian team members and recruited to participate in the São Paulo workshop. No private sector stakeholders were identified for recruitment in the Lima or Antigua Guatemala workshop. For each workshop, an effort was made to include 8–12 participants from each of the food and transport systems content areas (i.e., a total of 16–24 participants per workshop), and to include participants from diverse countries (with the exception of the São Paulo workshop, due to language constraints). Each participant targeted for recruitment was emailed an invitation letter describing the SALURBAL study and purpose of the CBSD workshops; follow-up emails were sent by team members in the country hosting each workshop.

### 2.3 General workshop structure

Each of the three workshops took place either during a full-day session (Lima) or a 1.5-day session (Antigua Guatemala and São Paulo). Workshops were conducted in the predominant language of the host country (i.e., Spanish or Portuguese). The structure of each workshop was guided by the facilitation guide produced by the workshop's CMT (please see the supplement for a sample facilitation guide). The project employed an iterative approach whereby the structure of each workshop was slightly altered based on context and lessons learned from preceding workshops, as described in the next section. In general, workshops were composed of an introductory session with a general presentation about SALURBAL and an overview of complex systems thinking, followed by a series of scripted activities that were led by a facilitation team (Hovmand et al., 2015b).

Each facilitation team included a convener, two community facilitators (i.e., one with domain expertise in food and one in transport), two modeler facilitators, at least four recorders, and 1–2 process coaches. Generally, the convener introduced the project at the beginning of the workshop and helped close the workshop. Scripted exercises were typically led by the modeler facilitator in collaboration with the community facilitator. The modeler facilitators also developed causal loop diagrams (CLDs) that aggregated and combined variables and feedback loops across multiple CLDs produced by sub-groups of participants (see Supplemental Figure S5). Other roles on the facilitation team generally provided support (e.g., the recorders took notes throughout each workshop that were later used to inform analytic decisions, as described below). Further details describing each of these roles can be found on Scriptapedia (Hovmand et al., 2015a).

The agenda for each workshop included a series of scripted activities (see Table 2). While the scripts provided a structure for the critical components of each activity, they also allowed enough flexibility for the facilitation team to make adaptations both in the planning phases immediately before, and on-the-fly during the course of any given activity. This was done to ensure that each script achieved its intended objectives. A Graphs Over Time script encouraged participants to think dynamically and to define the boundary of the problem under investigation (e.g., a prompt was to “please think of a factor that influences healthy eating in cities”). In the Causal Loop Diagramming script, the modeler facilitator asked sub-groups of 3–4 participants to build a causal loop diagram that explains the feedback structure that generates the dynamic behavior of one to two factors in the food system or transportation system, and how that factor can influence a healthy urban environment. During the Action Ideas script, participants were asked how they would intervene on the systems depicted by the CLDs, the size of the potential impact and feasibility of the proposed interventions, and whether there might be unintended or secondary consequences. Each participant completed every scripted activity, either as part of the full group or in parallel among sub-groups of participants.

**Table 2:** Summary agenda from community-based system dynamics workshops.

Activity	Artifacts Produced	Notes on Design Decisions	Lima (minutes)	São Paulo (minutes)	Antigua Guatemala (minutes)
General presentation	–		Yes (40)	Yes (40)	Yes (40)
Hopes & Fears	List of hopes and fears for the workshop		Yes (45)	Yes (45)	Yes (45)
Graphs Over Time	Graphs showing the trajectories over time of variables that influence healthy eating in cities and transport-related variables that influence health in cities; themes in variables; ranking of relative importance of variables	Conducted in parallel by food group and transport group	Yes (40)	Yes (40)	Yes (40)
Causal Loop Diagramming	2-3 CLDs describing variables and feedbacks via which food behaviors impact health in cities; 2–3 CLDs that describe transport behaviors that influence health	Conducted in parallel by food group and transport group	Yes (60)	Yes (60)	Yes (60)
Presentation of CLDs	–	Conducted in parallel by food group and transport group	Yes (30)	Yes (30)	Yes (30)
Model synthesis	Synthesis CLD	In Lima, facilitators conducted an initial synthesis of the small group CLDs during lunch and presented back to the full group for critique	Yes (60)	See “Day 2″	See “Day 2″
Causal Loop Diagramming 2.0	6 CLDs describing variables and feedbacks via which both food behaviors and transport impact health in cities	In São Paulo and Antigua Guatemala, participants conducted a second round of “Causal Loop Diagramming” in small “mixed” groups of participants with domain expertise in food and participants with expertise in transport	–	Yes (45)	Yes (45)
Presentation of CLDs 2.0	–	Conducted among the full group of all participants	–	Yes (45)	Yes (45)
Action Ideas	List of action ideas to improve food and transport behaviors, ranked by feasibility and potential impact	Conducted in parallel by food group and transport group	Yes (55)	See “Day 2″	See “Day 2″
Reflection (Day 1)	–		Yes (20)	Yes (30)	Yes (30)
Break for night			No	Yes	Yes
Day 2	–				
Welcome and review	–		–	Yes (20)	Yes (20)
Model synthesis	Synthesis CLD	In São Paulo and Antigua Guatemala, facilitators conducted an initial synthesis of the previous round of CLDs during the overnight break and presented back to the full group for critique	–	Yes (70)	Yes (70)
Presentation on leverage points	–		–	Yes (30)	Yes (30)
Action ideas	List of action ideas	Conducted in parallel by food group and transport group	–	Yes (45)	Yes (30)
Action ideas presentation	List of action ideas ranked in terms of impact and feasibility	Conducted among the entire group	–	Yes (45)	Yes (45)
Reflection (Day 2)	–		–	Yes (30)	Yes (30)

Note: CLD = causal loop diagram. Please see Scriptapedia for a more robust description of scripted activities (Hovmand et al., 2015b).

As shown in Table 2, many of the scripted activities produced artifacts. We primarily report on artifacts produced during the Causal Loop Diagramming script (i.e., CLDs) and the Action Ideas script (i.e., a list of action ideas). Although we do not describe artifacts produced in the Hopes and Fears or Graphs Over Time scripts, it is important to note that these scripts and the artifacts produced were critical to the workshops and supported subsequent scripts. For example, the Graphs Over Time script was used to help participants think about food and transport as dynamic variables that change over time. The variables identified during this script informed those that emerged in the CLDs developed in the subsequent script.

### 2.4 Differences between workshops

The CMTs made subtle changes to the activities and structure of the second and third workshops in response to lessons learned and participant feedback in the first workshop. The Lima workshop took place during a single day, but the workshops in São Paulo and Antigua Guatemala were extended to 1.5 days to allow participants more opportunities to work across sectors and refine their thinking together, as well as a night for participants to reflect on the knowledge gained during the first day. A key difference in the structure of the workshops is that in the Lima workshop there was a single causal loop diagram (CLD) workshop completed by small groups of food *or* transport domain experts. The São Paulo and Antigua Guatemala workshops included an additional CLD activity that was completed by small “mixed” groups of participants with domain expertise in food *and* participants with expertise in transport. The purpose of this combined CLD activity was to promote transdisciplinary thinking between the groups and to understand how stakeholders conceptualize connections between the systems driving food behaviors and transport. The São Paulo and Antigua Guatemala workshops also included additional time for the facilitation team to present the synthesized penultimate CLDs to participants, to receive feedback, and to modify the feedback structures to more accurately represent participants’ perceptions of the underlying dynamics.

Another important difference between workshops was that we slightly modified the Action Ideas script based on lessons learned from the preceding workshops, in order to facilitate further integration between the CLDs and the intervention ideas. In Lima, participants wrote their action ideas onto a piece of printer paper, but did not map the action ideas directly onto the CLDs. In São Paulo, participants had printouts of the CLDs that they could use individually to either visualize or trace out how an action idea would work within the context of the variables and relationships in the CLD. In the final workshop in Antigua Guatemala, participants worked with the modeler facilitator to add their action ideas directly onto the synthesis CLD produced by the group. This final iteration facilitated critical thinking regarding how interventions work within the context of a system, as well as group discussion because action ideas were “mapped onto” the synthesis CLD in real time while being projected for participants' review and feedback. These iterations in design were purposeful to facilitate and encourage more explicit consideration of how policies could work within the system of variables and feedback loops depicted in the CLDs. For example, consideration of the CLDs can help participants to identify leverage points for intervention in the system (i.e., places where a small change can produce a large shift in system behavior), as well as sources of resistance that may impact the intervention's effectiveness (Meadows, 2008).

### 2.5 Analysis

#### 2.5.1 Content analysis of variables

Three team members reviewed the CLDs created during all three workshops and conducted a content analysis to identify common themes in the variables in the CLDs. All three team members were from Latin America. One had deep methodological expertise in system dynamics modeling and domain expertise in transport. The other two had exposure to complex adaptive systems, primarily through participation in the 2.5-day training and planning session at the beginning of the project; one had domain expertise in transport, while the other had expertise in food. The purpose was to develop a qualitative understanding of the themes that emerged in each of the workshops, as well as to provide some general insight into the frequency with which variables with similar themes were present across workshops. Each team member used Qualtrics software to group all of the variables included in the final CLDs from the regional workshops into thematic piles. A separate team member then “labeled” the theme that emerged from each pile, compared the piles generated by each team member, and developed a list of the themes that emerged and their corresponding variables. The themes, labels, and variable lists were then presented back to the SALURBAL systems working group and feedback was solicited. No conflicts in labeling or theme identification were identified by the working group.

#### 2.5.2 Synthesis

After all workshops were completed, we conducted a multi-stage synthesis. The purpose was to produce a single “synthesis” CLD that, in the simplest way possible, captures the major variables and feedback loops that drive change over time in food behaviors and transport in Latin American cities, as identified by participants in the three workshops. Inputs to the synthesis included the final CLDs produced in each of the workshops, as well as reports and notes from each workshop. In total, there were four input CLDs: one transport CLD and one food system CLD from Lima, and one cross-domain CLD each, from São Paulo and Antigua Guatemala. Each CLD was produced during a series of iterative synthesis scripts that used CLDs produced by sub-groups of participants (i.e., during the Causal Loop Diagramming script). Please see Supplemental Figure S5 for a flow diagram showing how CLDs were combined in this study.

The aim of the first stage of the synthesis was to develop a list of the feedback loops in each input CLD, and to identify those feedback loops that were common across multiple CLDs. Three team members (one with content expertise in food and methodological expertise in complex systems; two others who were recorders during at least one of the workshops) began by identifying each of the major feedback loops and variables featured in the CLD produced during the São Paulo workshop; we started with this CLD because it had the most feedback loops. We then did the same for the other CLDs. As we identified each feedback loop, we qualitatively assessed whether there was a high degree of overlap between it and those previously identified (e.g., whether the dynamics described by a feedback loop from Antigua Guatemala were very similar to those identified in São Paulo). Key considerations in this assessment were text descriptions of each CLD created by workshop participants, as well as recorders’ notes from the workshops.

In the second step of the synthesis, the same three team members developed an “aggregate” CLD that included all unique feedback loops identified across the three workshops. In cases where feedback loops from multiple input CLDs described the same fundamental dynamics (as determined in the previous step of the analysis), the principle guiding the analysis was to reduce the variables and relationships in the CLD into the most parsimonious structure possible that captured the underlying dynamics. During this aggregation, we generally retained variable labels developed by participants. Notably, there were no disagreements about the directionality (i.e., + or -) of the relationships between common variables described by participants.

Lastly, in a third stage, three team members (one who participated in the previous round of synthesis, one with content expertise in transport, and one with methodological expertise in CBSD) conducted multiple stages of refinement in order to produce a “synthesis” CLD. The purpose of the synthesis was to develop a concise CLD with a reduced number of feedbacks that depicted fundamental dynamics described by participants during the workshops. At each stage, we compared the synthesis CLD with supporting information from the workshops (e.g., workshop notes, reports, and original artifacts) to ensure integrity and correspondence to the original intentions of workshop participants.

Concurrent with each stage of synthesis, we presented the initial versions of both the aggregate CLD and synthesis CLD to team members involved in the planning and implementation of the workshops; team members provided verbal and written feedback that we then used to guide revisions.

### 2.6 Institutional Review Board approval

The study protocol was reviewed by the Drexel University Institutional Review Board, which determined that the study did not consist of human subjects research. Investigators in Brazil received formal approval for the Brazil workshop in accordance with the requirements of their local Institutional Review Board.

## 3 Results

### 3.1 Objective E1: Stakeholder engagement

A total of 62 stakeholders participated in the three workshops, including 32 with expertise in the food systems domain and 30 in the transport systems domain (see Table 3). Latin American countries were broadly represented, with participants representing ten different countries. There was a fairly even mix of stakeholders from academia, civil society, and policymaking across all three regions. In Brazil, two stakeholders from the private sector also participated in the workshop.

**Table 3:** Participants in SALURBAL community-based system dynamics workshops.

	Lima, Peru	São Paulo, Brazil	Antigua Guatemala, Guatemala
**Total Participants**	17	24	21
**Content Area**			
Food	9	12	11
Transport	8	12	10
**Domain**^a^			
Academic	8	10	7
Civil Society	6	7	6
Policymaker	5	5	8
Private Sector	0	2	0
**Countries Represented**	Peru (11), Chile (3), Argentina (2), Brazil (1)	Brazil (24)	Guatemala (9), Mexico (5), Colombia (2), Costa Rica (2), Panama (2), El Salvador (1)

a Domain in São Paulo was self-reported by participants and could include multiple areas (e.g., academic *and* civil society).

Following each workshop, participants received a report and the facilitation guide that was used to implement the workshop in Spanish or Portuguese. The purpose was to provide a tangible deliverable via which participants could see some of the results of the time they committed to the project, to serve as a vehicle for continued engagement with SALURBAL, to facilitate collaboration between participants, and to facilitate continued systems thinking and use of these types of methods in participants’ own work. The report was prepared by the CMT and included background about the study, the explicit and implicit objectives guiding the workshops, an overview of participant information (i.e., number of participants by country and affiliation), the summary agenda, the CLD/s produced during the workshop, feedback loops and descriptions provided by participants during the workshop, action ideas, and reflections and insights that emerged from the workshop.

### 3.2 Objective E2: Gain experience in application of systems approaches and use of CLD

Both participants and members of our team gained experience in CBSD and use of causal loop diagrams to understand urban health. Fifteen team members participated in the two-day training in CBSD methods. Furthermore, 25 team members participated in the CMT for at least one of the three workshops. This included two methodological experts with experience in CBSD, who helped guide the process of designing the workshops. Thirty-nine team members participated in the facilitation team for at least one of the three workshops, meaning that they contributed to the implementation and execution of the workshops themselves. Roles played by team members during the workshop include conveners, facilitators, reflectors, process coaches, recorders, and observers, as well as five team members from the country hubs that provided logistical support. No formal evaluation was conducted to assess pre-versus post-workshop changes in knowledge or understanding dof systems approaches, use of CLDs, or impacts on problem understanding.

### 3.3 Objective I2: Learn about and expand mental models of stakeholders

In Supplemental Figures S1-S4, we present the four CLDs produced across the three workshops. This includes one food and one transport CLD produced in Lima, and one CLD that includes both systems produced in each of São Paulo and Antigua Guatemala. There were 98 variables across the four CLDs, including several that were common across more than one CLD. For example, all three workshops included consumption of ultra-processed foods (UPF) as an important individual-level behavior within the food system, and car use and congestion as key system-level behaviors within the transport system.

A key step in understanding how stakeholders view the systems that drive healthy eating and transport is identifying themes in the variables included in the CLDs. The research team identified eight themes via an inductive pile-sorting exercise, to which we assigned the following names: 1) health outcomes, 2) individual behaviors, 3) system behaviors and outputs, 4) time use, 5) built environment and access, 6) knowledge and attitudes, 7) policy and policymaking, and 8) social equity. In Table 4, we present these themes and variables.

**Table 4:** Variables included in causal loop diagrams produced in SALURBAL community-based system dynamics workshops, organized by domain.

Variable	L	A	S	Variable	L	A	S
*Health Outcomes*				*Built Environment & Access*			
o Health & health problems	X	X	X	• Food price & availability			
o Chronic disease		X		o Healthy vs ultra-processed food price	X	X	X
o Obesity	X	X		o Healthy vs ultra-processed food availability	X		X
o Health care costs		X		o Access to and capillarity of food retailers			X
*Individual Behaviors*				o Distance between food producers & consumers		X	
o Ultra-processed food consumption	X	X	X	o Urban, peri-urban, & organic agriculture			X
o Consumption of healthy food		X		o Financial interest	X		
o Nutrition			X	• Urban design			
o Physical activity	X	X	X	o Urban design			X
o Use of active transit		X	X	o City size		X	
o Use of public transit		X	X	o Peri-urban population	X		
o Car use	X			o Number of cars		X	
*System Behaviors & Outputs*				• Transportation infrastructure			
o Nutrition norms	X			o Mobility infrastructure			X
o Demand for unhealthy food	X			o Car infrastructure (e.g., highways, parking)	X		X
o Traffic congestion	X		X	o Public transit infrastructure		X	
o Pollution		X	X	o Space for pedestrians	X		X
*Time Use*				• Safety			
o Food preparation & consumption time	X	X	X	o Vehicle speed			X
o Commute time	X	X	X	o Road safety & accidents	X	X	X
o Free time	X		X	o Pedestrian safety		X	X
o Physical activity time			X	o Perceived safety of public spaces			X
o Screen time			X	• Other environment			
o Time at work	X			o Access to health services	X		
o Household duties/chores			X	*Policy & Policymaking*			
*Knowledge & Attitudes*				o Government policy & regulation	X		
• Knowledge & information				o Food industry lobbying	X	X	
o Food marketing	X	X	X	o Advocacy	X		X
o Dietary guidelines	X			o Political will	X		
o Nutrition literacy	X			o Policy proposal & implementation		X	
• Attitudes & values				*Social Equity*			
o Palatability of fresh food		X		o Social status	X		
o Social value of food			X	o Income	X		
o Commensality	X		X	o Gender equity & women in the labor force		X	
o Concern for health			X	o Women preparing meals		X	
o Social position associated with healthy vs ultra-processed foods			X				

Note: The “x” represents whether the variable was included in the causal loop diagram in a given workshop. L = Lima, A = Antigua Guatemala, S = São Paulo. Domains are italicized, sub-domains are in filled bullets, variables are in hollow bullets.

### 3.4 Objective I4: Identify common structures/drivers and variations across cities

**Fig. 1:**
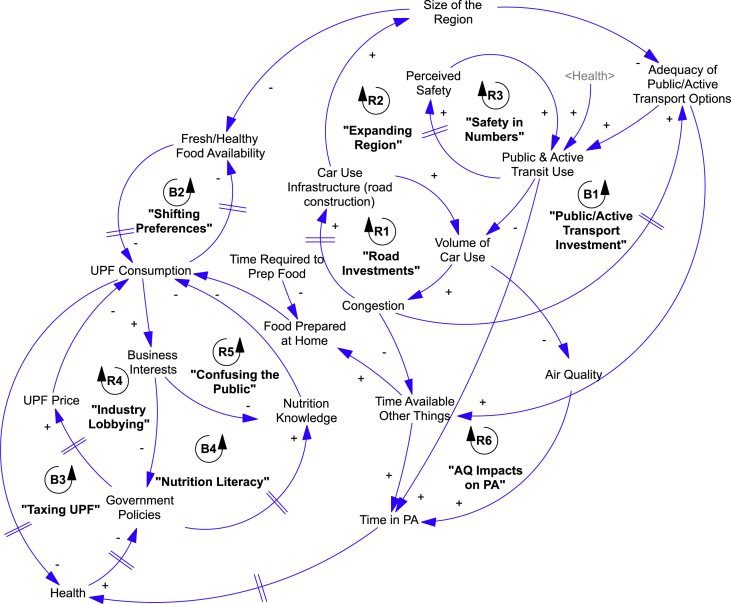
Synthesis causal loop diagram of the system that influences food behaviors and transport, based on three community-based system dynamics workshops in Latin American cities. Notes: Text that is bolded and in quotation marks is a feedback loop label; non-bolded text is a variable label. AQ = Air Quality. PA = Physical Activity. The presence of a “||” symbol on an arrow represents a time delay in the relationship between two variables. Variables in angle brackets (e.g., <Health>) are ghost variables or aliases that represent variables that appear elsewhere in the model. Figure generated in VensimPLE 7.2.

In Fig. 1, we present the synthesis CLD that includes the major feedback loops from CLDs from all three workshops. The synthesis CLD includes four balancing and six reinforcing feedback loops. In Table 5, we present a description of each feedback loop. The descriptions were developed by participants and were translated and lightly edited for clarity by the research team; the research team used these descriptions to create brief, informal names for each feedback loop.

**Table 5:** Description of feedback loops in a synthesis causal loop diagram based on three workshops to understand the systems that influence healthy diet, mobility, and transport in Latin American cities.

#	Feedback Loop	Workshop/s	Description
R1	Road investments to ease congestion	Lima	As car use and congestion increase, governments invest in construction of more roads and highways to ease congestion. The better infrastructure temporarily reduces congestion, but, over time, more drivers use the road and congestion eventually increases.
R2	Expanding region and car use	Antigua Guatemala, Lima	As cities increase their car use infrastructure, it becomes easier for commuters to live outside of the city. As the city grows, the public transit system becomes inadequate for more commuters, who then rely on private vehicles. The longer commutes and increased use of private vehicles increases congestion, which causes cities to invest in more car infrastructure; ultimately this leads to more urban sprawl.
R3	Safety in numbers	São Paulo, Antigua Guatemala	As the perceived safety of public spaces increases, more people will engage in active and public transit. More people on the street and engaged in active transit leads to greater perceived safety of public spaces.
R4	Industry lobbying	Lima	As the food industry gains more economic strength, they exert influence through lobbying and decrease political will to pass policies (like taxes) to reduce consumption of ultra-processed foods. For example, lobbying efforts could be used to impede passage of an excise tax to decrease consumption of ultra-processed foods.
R5	Misinforming the public	Lima, Antigua Guatemala	As food manufacturers sell more ultra-processed foods, their marketing budgets increase. This means that they can market even more widely, increasing the appeal of ultra-processed foods. These include efforts (e.g., advertisements, misleading labels) that reduce consumers' nutrition literacy by convincing them that ultra-processed foods are healthy.
R6	Impacts on Air Quality on Physical Activity	Antigua Guatemala	As city residents shift from high car use to increased use of active and public transit, air pollution decreases. Better air quality encourages people to engage in more outdoor physical activity, which reduces obesity and improves overall health. As the population becomes healthier and more fit, they use active and public transportation at even higher rates.
B1	Public/active transport investment	Lima, São Paulo	As car use and congestion increase, governments invest in improvement or expansion of the public and active transit infrastructure. Commuters respond to the congestion and improved infrastructure by using more public and active transit. This reduces car use and congestion.
B2	Shifting preferences	Lima, Antigua Guatemala	As consumption of ultra-processed food consumption increases, social norms towards foods change and people purchase and consume fewer fresh and healthy foods. Over time, food producers and retailers respond to the change in market demand by growing and selling fewer healthy foods. This reduced availability leads to even less consumption of healthy food and more reliance on ultra-processed foods.
B3	Taxing ultra-processed foods	Lima, São Paulo	Increased consumption of ultra-processed foods eventually leads to an increase in obesity and diet-related chronic disease. Eventually, the government may respond to declines in population health by imposing a tax on ultra-processed foods (e.g., Mexico, Chile), which leads to a decrease in their consumption.
B4	Nutrition literacy	Lima	The government may also respond to declines in population health by passing policies to improve the nutrition literacy of the population. This can include mandatory food labeling or development of dietary guidelines. As the population's nutrition literacy increases, preferences and consumption of ultra-processed foods declines.

There were also important differences in the variables and feedback loops described across workshops. For example, participants in Antigua Guatemala (please see Appendix Figure S4) described an important feedback structure linking gender equity in the labor force, gender roles, and time use. Specifically, participants posited that an increase in women in the labor force promotes gender equity, which can affect the distribution of household responsibilities between men and women. This reduces the pressure on women to be the sole preparer of food in the household and, in turn, contributes to more women entering the workforce. Conversely, in societies with a highly gendered distribution of household responsibilities, the constraints on women's time will prevent many from entering the workforce.

An example of a feedback structure unique to the São Paulo workshop was a reinforcing loop related to urban agriculture and pollution (please see Appendix Figure S3). As urban and peri-urban agriculture increase, air pollution will eventually decrease as carbon dioxide is removed from the air. Because ozone pollution causes oxidative damage to plants that reduces crop productivity (Lobell and Gourdji, 2012; Wilkinson et al., 2012), the decreased air pollution leads to more and more productive urban and peri-urban agriculture.

### 3.5 Objective I1: Put health and health equity on the agenda of policymakers who may not think their work influences health

Collectively, stakeholders identified clear connections between the food and transport systems, respectively, and health. As shown in Table 4, participants in all three workshops included at least one health outcome as a variable in their CLD. Specific variables within the health domain included general health and health problems, chronic disease, obesity, and health care costs.

While no variables explicitly mentioned health equity, stakeholders did describe feedback loops pertaining to social equity. One example is the feedback loop related to gender equity described above. A second example described by participants in the Lima workshop related to commuting patterns and residential segregation. Specifically, many of the poorest residents of Latin American cities live in peri-urban slums on the outskirts of cities. This increases travel times between these individuals’ homes and workplaces, which limits the number of hours they can work and, as a result, their income. As cities grow, these individuals are often pushed even further outside of the city center, which lengthens their commutes even further.

### 3.6 Objective I3: Identify policy priorities for improving health through transportation and food system intervention

Participants identified 52 action ideas to improve health through intervention in the food and transport systems (please see Table S1 in the Appendix for the list of action ideas). In general, about half of these action ideas consisted of policies or interventions within the domain of the built environment and access. For example, participants in the Lima and Antigua Guatemala workshops identified taxes on ultra-processed foods as a high-impact strategy to improve health. Participants in all three workshops also identified urban planning strategies via which cities can be redesigned to facilitate healthy choices, including by improving walkability and safety, conserving vital public spaces, increasing housing density, and promoting mixed-use planning so that people can access food sources, recreation facilities, and other resources within their own communities without using cars.

Participants in the Lima and São Paulo workshops identified several action ideas related to policy and the policymaking process. Several policies were intended to address specific barriers or challenges within the food and transport systems. Within the food system, this included legislation to explicitly recognize that vacant areas may be used for urban gardening, as well as creation of operating regulations for urban farms. Within the transport system, this included development of improved fuel standards. Action ideas also reflect stakeholders’ recognition of the importance of the policymaking process itself – one example is an idea to incorporate advocacy into public policy agendas for urban redesign. Participants in the Antigua Guatemala and Lima workshops identified several action ideas to improve nutrition knowledge and attitudes. These included policies related to comprehensible nutrition labeling, social marketing and nutrition education, bans on food marketing directed at children, and development of dietary guidelines.

## 4 Discussion

The study successfully achieved several of its objectives, particularly those related to stakeholder engagement, capacity building, and working with stakeholders to develop explicit representations (e.g., CLDs) of their mental models. In reflecting upon the planning and implementation of the workshops, our team identified several insights regarding both the process and our understanding of stakeholders’ perceptions of the systems through which food and transport systems impact health.

### 4.1 Process insights

A major emphasis of the SALURBAL study is to help develop the research infrastructure, partnerships, and expertise necessary to advance public health and health policy research within the Latin American context. The process of planning and implementing the CBSD workshops was a clear opportunity for capacity building, because most research team members, including those from both Latin American and U.S. institutions, had little practical experience with these methods but high interest in using them. The team-based approach to planning and implementing the workshops was a purposeful design decision on the part of the team members that were experienced in these methods. This design decision is an important aspect of the study, and likely impacted both the structure of the workshops and artifacts (e.g., causal loop diagrams, action ideas) produced. The approach allowed for a high number of team members to learn about and gain practical experience with the methods. Key contributors to team members' learning included the two-day training that took place at the study's outset, as well as the guided planning and implementation process in which at least one methodological expert worked with the core modeling and facilitation teams. Given that use of complex systems methods in public health is in its early stages, we believe that this type of facilitated learning model can be quite useful for diffusion of these methods.

Several members of the research team have leveraged the experience gained through the development and implementation of the workshops to utilize CBSD methods in other aspects of their work. One example is that team members from Colombia have used CBSD methods to investigate the potential effects of a new cable car project (i.e., TransMiCable) that connects a low-income, self-built neighborhood on steep hillsides in Bogotá to the main station. These investigators used a similar process and workshop design to understand how the TransMiCable implementation will affect a range of environmental and social determinants of health, healthy behaviors, and health outcomes.

A second insight related to the process of implementing the workshops is that our team was struck by participants’ ability to describe the structure and function of the food and transport systems in incredible depth with only minimal training related to complex system structures and CLD notation. As described earlier, participants were able to identify a broad range of feedback loops, as well as to articulate examples of how these loops function.

A third insight is that stakeholders viewed their participation in the workshops quite favorably and felt that the tools (e.g., CLDs, common language) and experiences to which they were introduced helped them to see the issues with which they work from a different perspective. For example, one of the stakeholders worked for a government agency and initially questioned why he was asked to participate in an academic workshop. By the end of the workshop, this participant mentioned the utility of the complex systems approach and told a facilitator that he planned to seek external funding to continue to use CBSD and systems thinking within the context of his agency's work. Participants also expressed their appreciation for the materials they received after the workshop.

Finally, the workshops were not devoid of challenges or resistance. In follow-up interviews, multiple participants reflected that the workshops allowed for disagreements (e.g., in the relationships between variables) and fostered productive conversations to find common ground and resolve these disagreements. For example, some participants created CLDs that had feedback loop structures that mirrored those included in the introductory presentation, others felt limited to only including variables for which data had been collected. Some participants struggled with how to represent differences between groups (e.g., gender) in the CLD, or with drawing graphs of variables over time, instead drawing graphs of correlations between variables. Another source of friction was related to disagreements between participants regarding key variables and relationships to including in CLDs. These types of challenges are common in CBSD because it is an interpersonal process that encourages participants to make their mental models explicit and negotiate a revised view of a problem that integrates new and different perspectives. The design and facilitation of workshops emphasized the creation of CLDs as boundary objects, which are tangible representations of dependencies and interactions that cross disciplinary, organizational or social lines (Black and Andersen, 2012). Use of CLDs and other visual representations as boundary objects can be used to turn disagreement and conflict into collaborative problem-solving discussions (Black and Andersen, 2012). We found that, as is common in many CBSD workshops, the work of developing models helped participants to embrace conflict and generate new insight and meaning. Some of the challenges and sources of resistance we encountered in the workshops were anticipated and discussed by the core modeling team and facilitation team prior to the first workshop, while others emerged during the first and second workshop and discussed in planning and training for the remaining workshops.

### 4.2 System insights

In reflecting upon the workshops and findings, one of the insights we found interesting is that stakeholders were more readily able than we had anticipated to identify connections between the systems driving food and transport behaviors. For example, CLDs in each of the workshops included time use variables in both the food and transport systems. Participants in Antigua Guatemala further described how the size of cities is interrelated to both food and transport. An increase in car ownership can contribute to urban sprawl and, as cities grow, the distance between food producers and consumers becomes greater. This is just one example of the complex and dynamic interrelationships between the pathways through which living in cities impacts the health of urban dwellers.

A second insight is that some of the feedback loops described by stakeholders may help explain secular trends in food behaviors and transport, as well as policy responses. An important secular trend in many countries, including several in Latin America, is increased UPF consumption over the last several decades (Monteiro et al., 2010; Popkin and Hawkes, 2016; Rivera et al., 2004). The synthesis CLD suggests several dynamic mechanisms that may have contributed to these increases. For example, as UPF consumption rises, the food industry can fund lobbying activities to stifle policy interventions and can purchase food advertising that promotes and normalizes UPF consumption (i.e., feedback loops R4 and R5) (Kassahara and Sarti, 2018; Rovirosa et al., 2017; Théodore et al., 2014). In contrast, enactment of government policies to promote healthy eating occurs over a longer time horizon and occurs only after serious health effects emerge (e.g., the obesity epidemic). These feedback loops may help to explain why expansive policies to improve healthy eating have recently passed in Peru (i.e., front-of-pack warning labels), Chile (i.e., front-of-pack warning labels and marketing regulations), and Mexico (i.e., sugar-sweetened beverages and junk food tax). These countries are fairly far along in the nutrition transition (Drewnowski and Popkin, 1997; Popkin, 1999, 2006), but evidence from Colombia and elsewhere suggests that the process of passing health-protecting legislation like excise taxes on sweetened beverages and tobacco taxes can be lengthy and require civil society engagement in advocacy, acquisition of evidence that includes monitoring of health consequences, estimation of relationships between variables, and policy evaluation (Moodie et al., 2013; Villar Uribe, 2017). This evidence can be used to inform public opinion, counter the effects of industry lobbying, and support policy decisions (Villar Uribe, 2017).

A third insight is that feedback loops may vary in their strength and implications in different contexts and starting conditions. For example, the synthesis CLD highlights two different mechanisms through which cities may respond to the problem of congestion. One of the feedback loops (R1) suggests that cities can attempt to ease congestion by investing in more and bigger infrastructure for cars (e.g., more and bigger roads and freeways). This type of investment, however, can contribute to urban sprawl. In contrast, cities can also respond to congestion by investing in active and public transit infrastructure with the goal of facilitating a shift in commuters’ transit mode choices and promoting healthy behaviors like physical activity (B1). Cities that are diffuse, that have previously invested in road infrastructure, or that have very high rates of car ownership may be less likely to invest in active and public transit infrastructure than cities that are more dense, have made these investments in the past, and that have less of a “car culture.” The importance of context and starting conditions can help explain why some cities (e.g., Guatemala City) invest predominantly in road infrastructure while others spend more on active and public transit infrastructure (e.g., Curitiba, Bogotá).

### 4.3 Integration with the SALURBAL study

In our view, it is premature to say whether we achieved some of the study's objectives. For example, the third explicit objective was to use insights from the workshops to identify and prioritize research questions and practice implications that our team will address using complex systems modeling in the future. Our team has begun development of two agent-based models: one to explore policies to improve active transit, the other to identify policy approaches to reduce consumption of ultra-processed foods. The CLDs produced in the workshops were among several inputs to the design of these models; however, the range of variables and relationships included in the CLDs was typically much broader than would fall within the boundary of any agent-based model that we could reasonably develop. That said, outputs from the workshops informed the outcomes and policy levers to be explored in each of the models. As the larger SALURBAL study continues to develop, an important step will be to further consider how insights from the workshops, including the CLDs, can be built upon through the use of other modeling efforts (e.g., formal system dynamics models).

The final implicit objective of the study was to test the waters for a potential simulation model, and to assess the potential for dissemination of complex systems approaches beyond academia. The workshops were extremely useful for demonstrating the high potential of participatory complex systems methods like CBSD modeling, but we did little to assess participants’ receptivity to simulation models. As the CMTs planned each of the workshops, it become clear that introducing stakeholders to simulation models would take substantial time out of the agenda that could be better used with scripted activities to introduce more general principles in systems thinking. Introducing stakeholders to simulation-based complex systems methods may take place in follow-up activities as SALURBAL evolves. This may, for example, include facilitated table-top exercises in which policymakers can use the agent-based models to explore policy counterfactuals.

### 4.4 Considerations

In considering our synthesis CLD and other findings, it is important to note that the purpose of the CBSD workshops was not to develop an authoritative depiction of the underlying systems that drive food behaviors and transport across Latin American cities or even within the specific cities in which the workshops were held. Some of the differences between variables and relationships between variables that we observed across workshops may be driven by the interests of the specific stakeholders present or chance and don't necessarily reflect regional differences in the real systems being depicted. Developing a representative depiction of the systems that drive health in a given Latin American city (or region), for example, might involve a series of workshops conducted serially with multiple cohorts of participants. If conducted across multiple cities (or countries), such an approach would allow for a more nuanced examination of commonalities and differences in the variables, feedback loops, and policies identified across different contexts. In contrast, our approach was designed to support our research team and stakeholder partners to view the systems with which we work from a broader perspective, and to generate insights to refine and prioritize future collaborative work. The artifacts (e.g., CLDs, action ideas) produced during the workshops reflect the unique knowledge and experiences of stakeholders and may be of high relevance in some urban contexts in Latin America and less relevant in others. Stakeholders were purposively recruited by SALURBAL team members based on team members' professional networks and knowledge of regional stakeholders with relevant domain expertise. As such, our findings may not be generalizable to other stakeholders or regions in Latin America.

Similarly, it is worth considering that important variables and feedback loops that link the food and transport systems to health and the environment may not be included in the CLDs or other artifacts produced during the workshops. For example, stakeholders in the three workshops did not focus on mechanisms via which food and transport behaviors contribute to and are influenced by global climate change. It is difficult to assess the extent to which the omission of these mechanisms reflect the structure of the underlying systems being explored, stakeholders’ unique perspectives regarding these systems, workshop design (e.g., structure of scripted activities, prompts, time constraints), or the model boundary (e.g., we used the Graphs Over Time script to explicitly set the boundary to include only factors that occur within cities).

We used an unscripted approach to develop the synthesis CLD (Fig. 1). The unscripted approach relied on artifacts and notes from each workshop to identify the main narratives variables and feedback loops. Facilitation team members from each workshop were also involved in developing and reviewing multiple iterations of the synthesis model. We acknowledge, however, that this process does create space for bias in filtering the variables and feedback loops included in the synthesis model. The only bias that we knowingly used was one that favored the inclusion of feedback narratives, as that is the basis of the SD approach. Due to time and budgetary constraints associated with facilitating workshops across multiple cities, we did not validate the synthesis model by systematically soliciting participants’ feedback. In future research, it is worth considering a study design that would employ series of workshops that would enable participants to review, consider, and refine a synthesis model in an iterative fashion and help to elucidate common and divergent system structures that emerge across varying urban contexts.

Finally, the action ideas developed by participants were an important set of artifacts produced during the workshops. As mentioned in the methods section, we adapted the “Action Ideas” script prior to the final workshop in Antigua Guatemala to ask participants to explicitly draw how each intervention would affect the variables and relationships included on the CLD produced by the group. In contrast, during the previous two workshops we asked participants to consider how each intervention would impact the system but did not ask them to explicitly draw these impacts on the diagram. As a result, we are unable to assess how each action idea may (or may not) influence the systems of variables and feedback loops elucidated by participants, or how the workshops and CLDs may have impacted participants’ thinking with respect to intervention approaches.

## 5 Conclusions

The CBSD workshops were a method for meaningful, early engagement of policy and practice stakeholders. The guided process of planning and implementing the workshops was a unique approach that helped build our diverse team members’ capacity for CBSD methods and deepen our capacity for systems thinking. The workshops were an important vehicle for introducing stakeholders to complex systems thinking, and generated artifacts and insights that are useful for understanding the mechanisms through which the food and transport systems impact health. We hope that this study can serve as a blueprint for other multi-disciplinary, stakeholder-engaged studies that use the tools of complex systems to examine drivers of urban health.

## Declarations of interest

None.

## Funding

The Salud Urbana en América Latina (SALURBAL)/Urban Health in Latin America project is funded by the Wellcome Trust [205177/Z/16/Z]. More information about the project can be found at www.lacurbanhealth.org. JDM received funding from the joint grant of Research Offices at the Universidad de Los Andes and the Universidad de Ibagué (project 19-528-ESP).
