# Supplementary material for: Using community-based system dynamics modeling to understand the complex systems that influence health in cities: The SALURBAL study
Source: Health Place. 2019 Nov;60:102215. doi: 10.1016/j.healthplace.2019.102215 (PMC6919340; doi:10.1016/j.healthplace.2019.102215)
Supplement: Multimedia component 2 [file mmc2.pdf]

What makes cities healthy, equitable and environmentally sustainable?
Lessons from Latin America

Lima, Peru Group Model Building Workshop

November 9, 2017

Workshop Agenda

Created: October 1, 2017
Last revised: November 2, 2017

What makes cities healthy, equitable and environmentally sustainable?
Lessons from Latin America

Universidad Peruana Cayetano Heredia
Lima, Peru
November 9, 2017

Objectives.....2

Modeling Team.....3

Participants: 5

Planning Logistics.....5

Agendas.....6

Group Model Building Scripts.....15

Objectives

Workshop Objectives (Explicit):

1. Bring diverse stakeholders into an initiative to promote healthy, equitable, and sustainable cities in Latin America
2. Gain experience in application of systems approaches in urban health problems and use of causal loop diagrams to identify and explore policy options
3. Participants will provide input that will help identify and prioritize research questions and practice implications to be pursued by the SALURBAL project using systems modeling in the future

Workshop Objectives (Implicit):

- Put health and health equity on the agenda of policymakers who may not think their work influences health.
- Learn about and expand mental models of stakeholders (academia, policymakers, banks, civil society) around transportation, food systems, and health
- Identify policy priorities for improving health through transportation and food system intervention and learn what is of value to stakeholders
- Identify common structures/drivers and variations across cities/contexts, and determine whether outputs of these workshops can inform the development of a simulation model
- Assess and test the waters for a potential simulation model/systems approach and dissemination beyond academia

Modeling Team

Core Modeling Team

The core modeling team (CMT) is responsible for the design of the workshop.

Role	Lima
Host Representative	
Modelers	
Familiarity with Stakeholders	
Scientific Objectives	
GMB Process	

Facilitation Team

The facilitation team is responsible for the delivery of the workshop itself. Facilitation team members may be a part of the core modeling team, or could be brought in for the facilitation only.

Role	Lima
Convener/Closer ***	
Community Facilitator	
Modeler Facilitator *	
Recorder	
Process Coach *	
Reflectors	
Production Coordinator ***	
Runners	
Wallbuilder **	
Observer	

Facilitation Team Roles

Meeting Convener/Closer: Primary responsibility for starting the session, introducing participants to the exercise, making sure that participants understand the purpose of the exercise within the context of their organization or community, and introducing the facilitators. Closer has primary responsibility for bringing the session to close and thanking participants for their time.

Community Facilitator: The community facilitator's primary responsibility is to extend their social capital to help the community accept and work with the modeler facilitator. This is a person who is familiar with the local or substantive knowledge of the problem being modeled and knows the local language and community norms in cross-cultural situations. The substantive expert/facilitator should have strong group facilitation skills, some exposure to system dynamics (e.g., through the planning process and training session or workshop), and have sufficient knowledge of the topic and/or community to anticipate and mediate conflicts that might arise within the group model building session.

Modeler Facilitator: Primary responsibility for system dynamics modeling and group model building process. This is a person who is trained in systems thinking/system dynamics model with expertise teaching and leading groups in the use of systems/thinking/system dynamics. The person should also have experience facilitating groups and leading group model building sessions. If the goal of the project is to develop a simulation model, it is expected that the modeler/facilitator also be an expert modeler and able to anticipate and address the variety issues that can arise in data and modeling.

Modeler: The modeler's primary responsibility is to build the system dynamics causal maps, models, and simulations. This is a person with expertise in system dynamics modeling and software (Vensim, IThink/Stella, etc.), formulating and entering equations, testing and analyzing the model, and running simulations for answering policy questions.

Note Taker/Time Keeper: Primary responsibility for taking notes about what is said in the workshop and notifying the facilitation team when time is short. It is overall very important to start and end on time as much as possible.

Process Coach: The process coach's primary responsibility is to observe the group process with attention to how participants are experiencing the session. This role requires someone who is able to reflect on the group process and accurately identify what is happening for participants based on observing their behavior and language. The process coach also plays an evaluation role and helps provide accurate feedback to the core modeling team about how the sessions are going. The process coach should be noticing when group dynamics begin to interfere with the process and identify potential solutions.

Production Coordinator: The production coordinator’s primary responsibility is to ensure that the information collected during the exercises, which includes diagrams, group model building scripts, agenda, pictures, notes, electronic versions of diagrams, etc., are collected, appropriately archived, and made available.

Reflector: Primary responsibility for helping the group reflect on what they have done so far and recognize the issues/insights that have been developed during the modeling. This role requires someone who can speak to the relevance of the activities and insights to a larger substantive context, or to system dynamics modeling more generally.

Runner: Primary responsibility is to be available to solve logistical problems as they emerge – including technical assistance, materials, etc. These people can also be on hand to support with wall building.

Wall Builder: The primary responsibility of the wall builder is to organize products from an exercise into thematic clusters, as well as to explain the clusters to the participants in order to elicit their feedback.

Participants:

Country	Stakeholder type	Name	Professional title	Institution	Expertise
Argentina	Subject matter expert				Food
Brazil	Policymaker				Food
Chile	Policymaker				Food
Peru	Policymaker				Food
Peru	NGO Rep				Food
Peru	Subject matter expert				Food
Peru	Policymaker				Food
Peru	NGO Rep				Food
Peru	NGO Rep				Food
Peru	Subject matter expert				Food
Peru	Dev Bank Rep				Food
Argentina	Subject matter expert				Transport
Chile	NGO/CSO / Subject matter expert				Transport
Chile	Biostatisticia n				Transport
Peru	Policymaker				Transport
Peru	Subject matter expert				Transport
Peru	NGO Rep				Transport
Peru	Policymaker				Transport
Peru	NGO Rep				Transport
Peru	NGO Rep				Transport
Peru	Policymaker				Transport
USA/ Colombia	Dev. Bank Rep				Transport

Planning Logistics

Space Requirements

- 1 room with 40 person capacity

- 2 breakout rooms with 15-20 person capacity
- Wall space for chart paper
- Projector screen & projector
- Food and drink (coffee, etc)

Materials Needed

- Digital projector
- Fat flip chart markers (at least one per participant)
- White printer paper
- Blue printer paper
- Yellow printer paper
- Blue painter's tape
- Flip charts
- Colorful 1-in dot stickers (the kind that are used in yard sales)
- Name tags

Agendas

Summary Agenda

**Activity**	**Duration**	**Time**
Participants Arrive	30 min	800-830
Welcome & Introductions	20 min	830-850
General presentation	40 min	850-930
Hopes & Fears	45 min	930-1015
Graphs Over Time (Break out rooms)	40 min	1015-1055
Dots (Break out rooms)	5 min	1055-1100
Break	15 min	1100-1115
Causal Loop Diagramming (Break out rooms)	1 hr	1115-1215
Presentations (Break out rooms)	30 min	1215-1245
Lunch	1.5 hr	1245-215
Model synthesis	1 hr	215-315
Break	15 min	315-330
Action Ideas (Break out rooms)	55 min	330-425
Dots (Break out rooms)	5 min	425-430
Reflection	20 min	430-450
Close	10 min	450-500

Anticipated Outputs

**Activity**	**Output**
Welcome	--
General presentation	
Hopes & Fears	List of hopes & fears
Graphs Over Time (Food Systems & Transportation)	Transport BOTGs<br>Food system BOTGs<br>Clusters/themes
Dots	Ranking of relative importance of BOTGs
Break	
Causal Loop Diagramming (Food Systems & Transportation)	Transport CLD x 2-3<br>Food Systems CLD x 2-3
Presentations	--
Lunch	
Model synthesis	2 synthesis CLDs;<br>Identification of common structures/variables
Action Ideas (Food Systems & Transportation)	List of action ideas, ranked by feasibility, potential impact
Dots	Ranking of importance of variables in synthesis CLDs
Reflection	Notes on key points
Close	

Detailed Agenda

**Time**	**Activity**	**Roles**	**Description**
7:30-8:00	Room Setup		
8:00-8:30	Participants arrive		Folks will arrive
8:30-8:50	Welcome & Introductions<br><br>*Apertura y bienvenida*	Convener:	Key points:<br>• Introduction of facilitation team<br>• Introduction of participants<br>• Summary of goals and agenda for the day<br>• *Presentación del equipo de facilitación y sus roles*<br>• *Revisión de los objetivos*<br>• *Revisión de la agenda*
8:50-9:30	Presentation	SALURBAL :<br><br>Systems approaches:	Potential talking points:<br>• Latin American health in cities<br>• Integrating research and policy<br>• Systems approaches in general<br>• Examples of GMB in other contexts<br>Goal: communicate the dual benefit of this workshop for research and for stakeholders themselves

<table>
<tr><td>9:30-
10:15</td><td>Hopes & Fears

Aspiraciones y preocupaciones</td><td>Community facilitator:

Wallbuilder:</td><td>Community facilitator asks participants to write hopes and fears for the workshop on pieces of blue (fear) and yellow (hope) paper. She highlights that one participants should write one hope or fear per paper.
Gives 5 minutes to write as many hopes and fears as can think of.
With 1 minute left, ask participants to stack their hopes and their fears with the most important ones on top.
After 5 minutes, the facilitator asks someone to share 1 hope and 1 fear.

Modeler facilitator will take the hopes and fears and cluster them on the wall.
After 1-2 rounds of sharing, modeler facilitator will summarize the hopes and fears, highlighting thematic clusters, and asking for additional observations.

Example hopes and fears:
Preocupaciones necesarias:
Gente entrando y saliendo del taller para atender otros asuntos
Perdemos mucho tiempo en los recesos porque la gente se tarda en regresar
Aspiraciones necesarias:
La opinión de todos tiene el mismo peso y todos pueden participar abiertamente</td></tr>
<tr><td>1015-
1020</td><td>Move to breakout rooms</td><td></td><td></td></tr>
<tr><td>10:20-
10:55</td><td>Graphs Over Time

(split food systems & transportation into separate rooms)</td><td>Food group
Modeler facilitator:
Wallbuilder:

Transport group
Modeler facilitator:

Wallbuilder:</td><td>With each group (food & transport), the Modeler facilitator introduces the graphs over time by highlighting that it’s easy to come up with explanations about why we behave the way we do, but we need to really think deeper. What’s true today may not have been true before...He provides 2 examples. The examples will highlight two criteria: demonstrating multiple time horizons, and demonstrating tangible and intangible variables.
Options (to be confirmed Wednesday 8 Nov):
Endangered species
Coffee consumption
Sleep
Energy level
Each modeler facilitator will ask participants in their respective groups to draw as many graphs over</td></tr>
</table>

			time as they can in the next 5 minutes. The prompts used to facilitate this activity will be as follows:<br><br>***Food systems group PROMPT:***<br><br>“Por favor piense en un factor que influencie la alimentación saludable en las ciudades.<br><br>En cada hoja de papel dibuje la trayectoria, a través del tiempo, de un factor que influencie la alimentación saludable en las ciudades. Por favor incluya en la misma gráfica, una trayectoria que usted aspira que ocurra y una trayectoria la cual le preocupa que ocurra.”<br><br>***Transport group PROMPT:***<br>“Por favor piense en un factor de la movilidad y del transporte que tenga influencia sobre la salud de las ciudades<br><br>En cada hoja de papel dibuje la trayectoria, a través del tiempo, de un factor de la movilidad y del transporte que tenga influencia sobre la salud de la ciudades. Por favor incluya en la misma gráfica, una trayectoria que usted aspira que ocurra y una trayectoria la cual le preocupa que ocurra.”<br><br>Participants have **5 minutes** to draw the graphs, but this can be monitored and extended/shortened by a few minutes if necessary. At 4 minutes, a **1-minute warning** is given and they are asked to begin stacking their graphs over time with the most important/favorite on top and least important/least favorite on the bottom.<br><br>Modeler facilitator then calls a stop and then goes around using a nominal group technique where each person identifies their top graph over time and explains the graph, which is handed to Wallbuilder (who clusters the behavior over time graphs (BOTGs) on the wall.<br><br>In their respective groups, community facilitators then review the clusters and themes, highlighting uncertainty in her choices, and asks participants if there are any changes to be made in where each graph is situated.

10:55-<br>11:00	Dots<br>(split food systems & transportation into separate rooms)	Modeler Facilitator:	Modeler facilitator asks participants to take the dots they have in their tables and vote for which are the most important drivers of community trends over time. They can vote 5 times for the same graph, or one for each. Colors don't matter.
11:00-<br>11:15	Break		
11:15-<br>11:30	Introduction to CLDs<br>*Introducción a mapas causales*<br><br>(split food systems & transportation into separate rooms)	Modeler Facilitator:	First, the facilitator will tell a story of chickens reproducing, population growth, overcrowding, and injuries<br><br>Then, the facilitator will present 2 graphs over time to describe these dynamics – of chicken population growth and stabilization, and of injuries.<br>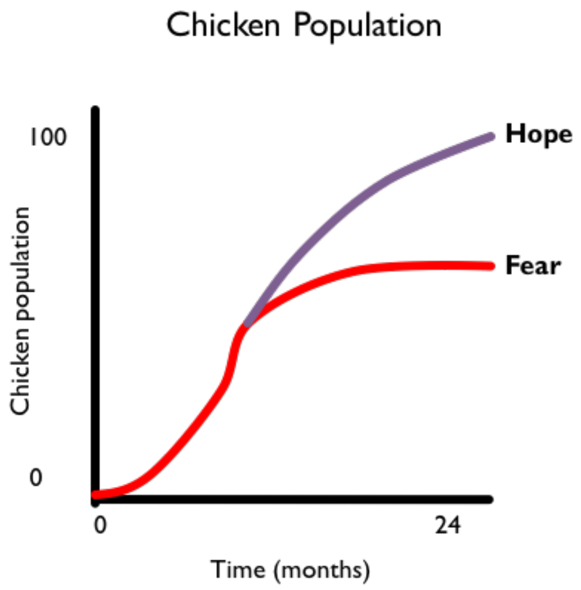

			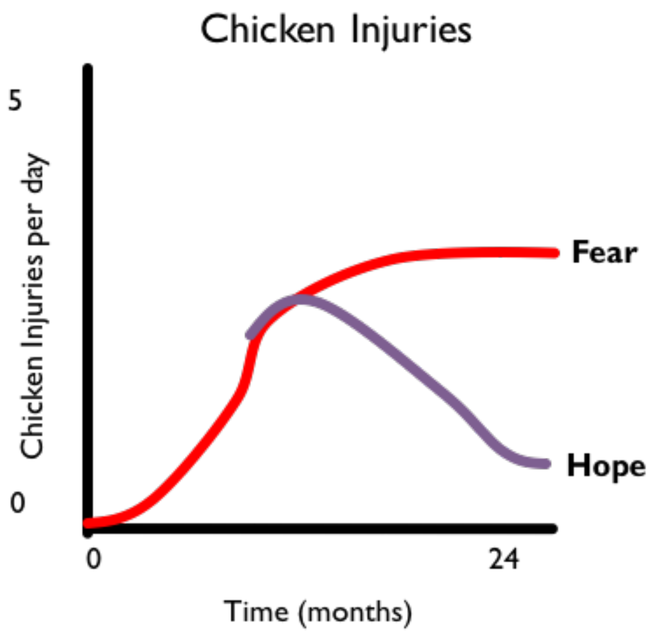<br>The facilitator then describes a set of feedback loops as a dynamic hypothesis of a structure that may create this behavior<br>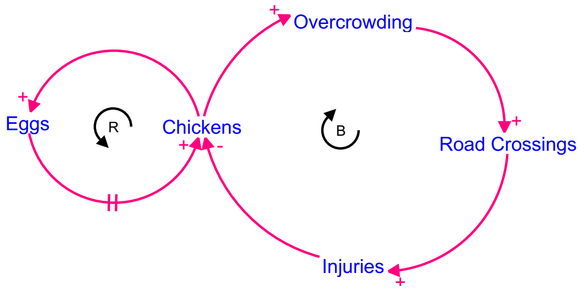<br>The presentation will highlight:<br>1. polarity,<br>2. variable definition<br>3. feedback<br>4. Delays<br>The facilitator emphasizes that the CLD is a hypothesis about the structures that could create the behavior. He then asks if there are other factors or structures, and can add on other sources of injuries, other factors that reduce eggs, etc.
11:30-12:15	Causal Loop Diagramming<br><br>*Trabajo en grupos: mapas causales*	Modeler Facilitator:<br><br>Community Facilitator:	Within the food systems and transport groups, people will randomly be assigned (by counting off) to one of 2-3 subgroups (each made up of 3-4 people).<br><br>Each subgroup will work on building their own causal loop diagram. Diagramming by the food groups will be facilitated by the prompt:

	(split food systems & transportation into separate rooms)		**Build a causal loop diagram that explains a hypothesis of the food systems/transportation system factors that influence a healthy urban environment.**<br><br>While groups work, the facilitation team will circulate, asking questions and providing guidance if folks are stuck.<br><br>With 5 minutes left Modeler facilitators give a 5-minute warning and ask folks to focus on consequences of target behavior, closing feedback loops.
12:15-12:45	Presentations	Community Facilitator:	Teams will present their models to the larger group, focusing on highlighting feedback loops and key stories.<br><br>*[Meanwhile modelers begin synthesizing CLD with the aim of creating two causal loop diagrams: 1 for food systems, and one for transport.]*
12:45-2:15	Lunch		*[Modelers continue synthesizing CLD]*
2:15-3:15	Model Synthesis	Modeler Facilitator:<br><br><br>Community Facilitator:	Modeler facilitator will describe how over lunch, the facilitation team reviewed the models and created a united conceptual model, one for food and one for transport, that links all of the CLDs that were developed in the first session. Then, one at a time, the modeler facilitators will project the synthesized causal loop diagram (created using Vensim) for food systems and transport.<br><br>Modeler facilitator then describe how the CLDs were constructed using the input of the morning's CLD's, highlighting key stories and ideas.<br><br>The Modeler facilitator then leads a conversation with the team to review whether the model accurately captures the stories of the group, or whether there are new perspectives to be included.<br><br>Community facilitator will facilitate conversations among participants and ask clarifying questions.<br><br>Modeler facilitator will make revisions to the model based on proposed changes. The goal is to converge on a consensus view of the problem from a feedback perspective. We will revise the model until no one has adjustments or disagreements to add.

<table>
<tr><td></td><td></td><td></td><td></td></tr>
<tr><td>3:15-
3:30</td><td>Break</td><td></td><td></td></tr>
<tr><td>3:30-
4:25</td><td>Action Ideas – split Transportation & Food systems

Identificar propuestas de intervenciones e innovaciones

(split food systems & transportation into separate rooms)</td><td>Community Facilitators:

Wallbuilder:</td><td>The community facilitator presents on different types of system interventions, from accelerating feedback loops to creating information flows, highlighting that a change to a parameter is often the easiest but lowest leverage change we can make.

Community facilitator asks participants to think about ways we can intervene in this system. He asks teams to come up with their top 4 system interventions, written on a white piece of paper, and plan to map these onto the model.

At the end of 5 minutes, the facilitator asks for a volunteer group to share.

The first group should describe how they would intervene in the system, how it would impact system performance, unintended or secondary consequences. They will then propose where to put the intervention on the matrix of feasibility and potential impact.

The wallbuilder places the action idea on the wall based on this recommendation.
Introducción a la identificación de intervenciones (15 minutos)
Ahora trabajarán en los mismos grupos para identificar intervenciones e innovaciones para influenciar los resultados de los mapas
Dibujar cada intervención e innovación en el mapa y en una post-it
Trabajo en grupos (30 minutos)
Presentación de ideas y colocación de intervenciones en cuadrantes (45 minutos)
Eje X: Impacto potencial
Eje Y: Dificultad de implementar (costos, complejidad, etc.)</td></tr>
<tr><td>4:25-
4:30</td><td>Dots</td><td>Community Facilitator:</td><td>Community facilitator distributes 6 blue dots to transport experts and yellow dots to food experts.</td></tr>
</table>

			Community facilitator then asks participants to take the dots they have on their tables and vote for the **most important factors** shaping health in cities. They can vote 6 times for the same factor, or one for each.<br><br>**Focus on: Most important factors**
4:30-4:50	Reflection<br>*Reflexiones*	Reflectors:	Reflectors will reflect on the day's activities, focusing on the perspectives of:<br>1. Lima/Peruvian stakeholders<br>2. Research perspective,<br>3. Modeling perspective.
4:50-500	Closing	Closer:	

Group Model Building Scripts

The following scripts were used in the group model building workshop. Scripts are structured small group exercises used in group model building. These scripts are compiled in a wikibook called Scriptapedia which is intended to be a freely distributed book and easily edited to support the creation of new scripts, discussion of what works and what doesn't, and internationalization of group model building practice. Scriptapedia is available at https://en.wikibooks.org/wiki/Scriptapedia.

License

Scriptapedia is shared under the Creative Commons Attribution-ShareALike 3.0 Unported License. Anyone is free to share (copy, distribute, and transmit the work) and remix (adapt the work) under the following conditions: (1) users must attribute the work in a manner specified by the author of the content, but not so in a way that suggests that they endorse or are using adaptation, and (2) if a user alters, transforms, or builds upon another author or authors' works, users can distribute the resulting working only under the same or similar license as this one. With this understanding, any of the above conditions can be waived if the user gets permission from the copyright holder. If any or all of the elements of the work is already in public domain under applicable law, that status is in no way affected by the license. In no way are any of the following rights affected by the license: (1) users fair dealing or fair use rights, or other applicable copyright exceptions and limitations; (2) author's moral rights, and (3) rights other persons may have either in the work itself or in how the work is used, such as publicity or privacy rights. For any reuse or distribution, the user reusing or distributing the Molecule Library and its contents must make clear to others the license terms of this work. The best way to do this is by copying this page or linking to the Creative Commons Attribution-ShareALike 3.0 Unported License website.
