# Supplementary material for: Using community-based system dynamics modeling to understand the complex systems that influence health in cities: The SALURBAL study
Source: Health Place. 2019 Nov;60:102215. doi: 10.1016/j.healthplace.2019.102215 (PMC6919340; doi:10.1016/j.healthplace.2019.102215)
Supplement: Multimedia component 3 [file mmc3.docx]

**SUPPLEMENTAL INFORMATION**

| **Table S1: Action ideas produced by participants in SALURBAL community-based system dynamics workshops, organized by domain** |
| --- |
| *Built Environment & Access* |
| Prohibit sales of sugary drinks in schools (L) |
| Microcredit and credit assistance for medium-sized farms (S) |
| Public transit subsidies (L) |
| Taxes on processed food (L) |
| Taxes on junk food and SSB (A) |
| Supply-side public policies to increase capillarity of healthy food distribution (S) |
| Using integrated infrastructure to distribute fresh foods (S) |
| Availability and regulation of sustainable food production (L) |
| Organizing weekend markets at public schools to sell healthy foods (S) |
| Markets and selling points on transportation lines (S) |
| Orchards (huertos) in schools and workplaces (L) |
| Sustainable agriculture (A) |
| Development of healthy processed foods (L) |
| Promoting safe streets (L) |
| Use of traffic lights to reduce congestion (L) |
| Intervention to promote walking and biking as the default option. Pedestrian and bike transit infrastructure would displace car infrastructure as the central component of transit infrastructure. Example: sustainable transport that emphasizes the social component. (L) |
| Urban planning to promote shorter travel distances by giving people access to food sources, recreation, health, and other necessities within their neighborhoods (L) |
| Strengthen social control (revising master plans for sustainable cities) (S) |
| Recovery of public space (L) |
| Housing density policies (L) |
| Urban design incentives (S) |
| Walkability of cities (A) |
| Policies to improve traffic management and street design (L) |
| Revising city master plans and design instruments (S) |
| Systemic changes to make public transit the first choice before travel via private vehicle (L) |
| Policies and incentives to increase bicycle use (e.g., Bogota provides free bicycle use one day per month) (L) |
| Government policies to increase safety (A) |
|  |
| *Individual Behaviors* |
| Promote use of public transport (L) |
| Promote other forms of travel (e.g., walking, bicycling)(L) |
| Promoting cycling tours between vegetable gardens, small markets, and healthy eating places (S) |
| Government policies to increase active transport (A) |
|  |
| *System Behaviors & Outputs* |
| Convergent solutions across fields (i.e., multidisciplinary approaches that link multiple sectors that influence health, such as transport and ecosystem management) (S) |
| Integration of modes of transport (e.g., bus rapid transit and metro in Bogotá) to reduce congestion (L) |
|  |
| *Time Use* |
| Extend the school lunch period (L) |
| Develop fast and healthy recipes (L) |
|  |
| *Policy & Policymaking* |
| Urban legislation recognizing use of vacant areas for urban gardens (S) |
| Policy of urban planning and urban conservation that prohibits construction of mega-structures in order to improve public space and conserve neighborhoods (L) |
| Ultra-processed food regulations (S) |
| Advocacy that articulates goals to bring together different initiatives and areas (S) |
| Incorporating advocacy into public policy agendas for urban redesign (S) |
| Decentralized government (e.g., bus rapid transit began as result of a local government and social policies) (L) |
| Advocacy for food and transport (S) |
| Improve fuel and gas standards (L) |
| Creation of operation regulations for urban farms (S) |
|  |
| *Knowledge & attitudes* |
| Front of product nutrition labeling (A) |
| Healthy diet and nutrition education (A) |
| Nutrition promotion programs in workplace (L) |
| Social marketing (L) |
| Consumer nutrition education (L) |
| Regulation of food marketing targeted to children (L) |
| Dietary guidelines developed via consensus (L) |
| Mandate inclusion of clear nutrition information on food packaging (L) |
| Note: Letters in parentheses refer to the workshop in which the variable was included in the causal loop diagram. L = Lima, A = Antigua Guatemala, S = São Paulo. |

**Figure S1: Causal loop diagram of the system that influences food behaviors and health, produced by participants in a community-based system dynamics workshop in Lima, Peru. The presence of a “||” symbol on an arrow represents a time delay in the relationship between two variables. Figure generated in VensimPLE 7.2.**

**Figure S2: Causal loop diagram of the system that influences transport and health, produced by participants in a community-based system dynamics workshop in Lima, Peru. The presence of a “||” symbol on an arrow represents a time delay in the relationship between two variables. Figure generated in VensimPLE 7.2.**

**Figure S3: Causal loop diagram of the system that influences food behaviors and transport, produced by participants in a community-based system dynamics workshop in São Paulo, Brazil. Notes: The presence of a “||” symbol on an arrow represents a time delay in the relationship between two variables. Variables in angle brackets (e.g., <Advocacy>) are ghost variables or aliases that represent variables that appear elsewhere in the model. Figure generated in VensimPLE 7.2.**

**Figure S4: Causal loop diagram of the system that influences food behaviors and transport, produced by participants in a community-based system dynamics workshop in Antigua Guatemala, Guatemala. Notes: The presence of a “||” symbol on an arrow represents a time delay in the relationship between two variables. Figure generated in VensimPLE 7.2.**

**Figure S5: Flow chart of development and synthesis of causal loop diagrams**

| **Table S2: Complex systems terms and definitions** | |
| --- | --- |
| **Term** | **Definition** |
| System dynamics | System dynamics is the use of informal maps and formal models with computer  simulation to uncover and understand endogenous sources of system behavior (Richardson 2011). |
| Community-based system dynamics | An approach to understanding how stakeholders perceive the variables, relationships, and feedback loops that comprise a complex adaptive system and that prioritizes building stakeholders’ capabilities in the use of tools from the field of system dynamics (Hovmand 2013 and 2014). |
| Causal loop diagram | Causal maps that provide a broad view of the different components of a system, including major subsystems and how these are related through multiple feedback loops (Brennan et al. 2015). |
| Mental models | A cognitive representation of a real dynamic system (Doyle and Ford 1998; Hovmand 2014). |
| System | A set of elements or parts that is coherently organized and interconnected in a pattern or structure that produces a characteristic set of behaviors, often classified as its ‘function’ or ‘purpose’ (Meadows 2008). |
| Reinforcing feedback loop | An amplifying or enhancing feedback loop, also known as a ‘positive feedback loop’ because it reinforces the direction of change. These are vicious cycles or virtuous cycles, depending on whether the outcome is detrimental or desirable (Meadows 2008). |
| Balancing feedback loop | A stabilizing, goal-seeking, regulating feedback loop, also known as a ‘negative feedback loop’ because it opposes, or reverses, whatever direction of change is imposed on the system (Meadows 2008). |
